# Organ on a Chip: A Novel *in vitro* Biomimetic Strategy in Amyotrophic Lateral Sclerosis (ALS) Modeling

**DOI:** 10.3389/fneur.2021.788462

**Published:** 2022-01-17

**Authors:** Babak Arjmand, Shayesteh Kokabi Hamidpour, Zahra Rabbani, Akram Tayanloo-Beik, Fakher Rahim, Hamid Reza Aghayan, Bagher Larijani

**Affiliations:** ^1^Cell Therapy and Regenerative Medicine Research Center, Endocrinology and Metabolism Molecular-Cellular Sciences Institute, Tehran University of Medical Sciences, Tehran, Iran; ^2^Health Research Institute, Thalassemia, and Hemoglobinopathies Research Center, Ahvaz Jundishapur University of Medical Sciences, Ahvaz, Iran; ^3^Endocrinology and Metabolism Research Center, Endocrinology and Metabolism Clinical Sciences Institute, Tehran University of Medical Sciences, Tehran, Iran

**Keywords:** amyotrophic lateral sclerosis, iPSC, microfluidics, motor neurons, organ on a chip

## Abstract

Amyotrophic lateral sclerosis is a pernicious neurodegenerative disorder that is associated with the progressive degeneration of motor neurons, the disruption of impulse transmission from motor neurons to muscle cells, and the development of mobility impairments. Clinically, muscle paralysis can spread to other parts of the body. Hence it may have adverse effects on swallowing, speaking, and even breathing, which serves as major problems facing these patients. According to the available evidence, no definite treatment has been found for amyotrophic lateral sclerosis (ALS) that results in a significant outcome, although some pharmacological and non-pharmacological treatments are currently applied that are accompanied by some positive effects. In other words, available therapies are only used to relieve symptoms without any significant treatment effects that highlight the importance of seeking more novel therapies. Unfortunately, the process of discovering new drugs with high therapeutic potential for ALS treatment is fraught with challenges. The lack of a broad view of the disease process from early to late-stage and insufficiency of preclinical studies for providing validated results prior to conducting clinical trials are other reasons for the ALS drug discovery failure. However, increasing the combined application of different fields of regenerative medicine, especially tissue engineering and stem cell therapy can be considered as a step forward to develop more novel technologies. For instance, organ on a chip is one of these technologies that can provide a platform to promote a comprehensive understanding of neuromuscular junction biology and screen candidate drugs for ALS in combination with pluripotent stem cells (PSCs). The structure of this technology is based on the use of essential components such as iPSC- derived motor neurons and iPSC-derived skeletal muscle cells on a single miniaturized chip for ALS modeling. Accordingly, an organ on a chip not only can mimic ALS complexities but also can be considered as a more cost-effective and time-saving disease modeling platform in comparison with others. Hence, it can be concluded that lab on a chip can make a major contribution as a biomimetic micro-physiological system in the treatment of neurodegenerative disorders such as ALS.

## Introduction

Amyotrophic lateral sclerosis, also known as Lou Gehrig's disease or motor neuron disease, is a neurodegenerative disorder that affects the motor nerve cells and eventually renders patients paralyzed. The progressive degeneration of motor nerve cells is one of the first complications associated with amyotrophic lateral sclerosis (ALS) that may trigger by either one or both genetic and environmental risk factors. As the gradual loss of motor neurons continues, it can lead to progressive deterioration in muscle functioning. If the muscle paralysis spreads throughout the body, it can lead to mobility impairments and even interfere with swallowing, speech, and breathing as well (1, 2).

Amyotrophic lateral sclerosis (ALS) is classified into two types: sporadic (sALS) and familial (fALS). According to studies, fALS accounts for a small percentage of patients (about 5–10% of cases) compared to sALS (about 90–95% cases) (3, 4). Clinical manifestations are common in both sALS and fALS (3) but initiating factors involved in these two types of ALS are different. According to studies, both genetic (5) (e.g., C9ORF72, SOD1, TARDB, and FUS genes) (6, 7) and environmental factors (dietary habits, body mass index (BMI), smoking, alcohol consumption, etc.) are proposed as two main factors involved in ALS pathogenesis (Table 1) (5, 8, 10, 13).

**Table 1:** Environmental factors involved in ALS pathogenesis.

**Environmental factor**	**Description**	**References**
Age	•The incidence rate of ALS is higher at older ages.	(8)
Gender	•The risk of ALS is higher in males compared to females.	(5)
Geographic region	•Genetic variation and diverse environmental factors exposure in different geographic regions can lead to different incidence rates. • In developed countries the prevalence is higher compared to less developed countries due to some reasons such as: 1) High exposure rate with some risk factors like environmental toxins in developed countries 2) Less diagnosis and evaluation of the disease in less developed countries	(5)
ALS Spatial clustering	•Eco-epidemiological studies imply that different ALS spatial clustering observed in diverse geographical areas due to: 1) Proximity to ecosystems containing beta methyl-amino-alanine (BMAA) neurotoxin-producing cyanobacteria 2) Water quality in terms of high total nitrogen (TN) for algae growth, chlorophyll-a (Chl-a) for cyanobacteria growth, and low Secchi depth (SD) (Criteria for water Transparency)	(9)
Dietary habits	•Having a high-fat and glutamate diet can be a potential factor in the high risk of ALS development.	(8)
Body mass index (BMI)	•Lower BMI condition may lead to a rise in ALS development risks.	(10)
Smoking	•Tobacco smoking can increase the risk of ALS development by its significant role in oxidative stress, inflammation, and neurotoxicity.	(10)
Alcohol consumption	•Although excessive alcohol use can play a role in ALS development increase, some lines of evidence indicate that alcoholic beverages may be as a barrier to the progression of ALS due to their antioxidant properties.	(10)
Physical activity	•Vigorous-intensity exercises and violent professional sports can lead to some adverse effects on ALS development. However, low-intensity and light exercise can slow down or prevent neuron degeneration.	(10, 11)
Personal and social activities	•High brain activity (e.g., reading a book, learning a new language, writing, painting) for more than 20 minutes a day can have a significant effect on preventing ALS risks.	(10)
Psychological stress	•A few studies have suggested a link between psychological stress and ALS incidence. Therefore, this case needs further investigation.	(8, 12)
Trauma	•Exposure to physical trauma (especially head trauma) can have a major contribution to ALS development.	(5, 10)
Electric shocks and magnetic fields	•Exposure to electric shocks and electromagnetic fields can be mainly associated with degeneration of nerve cells.	(13)
Heavy metals	•Exposure to heavy metals (e.g., selenium, mercury, and lead) may (certainly not) have contributed to the degeneration of nerve cells.	(5, 13)
Chemical compounds	•Exposure to different types of pesticides can have a major contribution to neurological disorders.	(5, 13)
Viruses	•There may be a link between some viral infections such as enterovirus (EVs) and retroviruses (like HIV) with ALS clinical condition.	(14–16)
Fungi	•According to evidence, in some cases, the ALS onset may be due to some fungi-produced neurotoxic mycotoxins. To date, the infectivity and neurotoxicity of several fungi such as Macrocyclic trichothecenes, Fumonisin B1, and Ochratoxin A have been studied in ALS pathogenesis. But it is thought that mutations in some of the ALS-related genes may predispose the body to fungal infections and finally result in immune systems weakness. However, this case needs further investigation.	(17)
Bacteria	•Some studies have revealed that several bacteria such as *Sphyngomonodales, Cutibacterium acnes, Corynebacterium sp, Fusobacterium nucleatum, Lawsonella clevelandesis, Streptococcus thermophiles, Burkholderia species, Firmicutes, Bacteriodetes, Actinomycetales, Burkholderiales, Rhizobiales, Actinobacteria, Proteobacteria, and even Xanthomonadales* may be related to ALS pathogenesis.	(18)
Microbiota	•Gut microbiota can have either a toxic or protective role in different patients. For instance, some evidence implies that clinically, in ALS conditions, an imbalance of the gut's microbial community (e.g., *Butyrivibrio fibrisolvens* and *Firmicutes* reduction) and intestinal epithelium permeability augmentation can be observed. In contrast, the existence of some bacteria such as *Akkermansia muciniphila* can have beneficial effects on ALS patients.	(19, 20)

*ALS, Amyotrophic lateral sclerosis; BMAA, Beta methyl-amino-alanine; BMI, Body mass index; Chl-a, Chlorophyll-a; EVs, Enterovirus; SD, Secchi depth; TN, Total nitrogen*.

**Figure 1:**
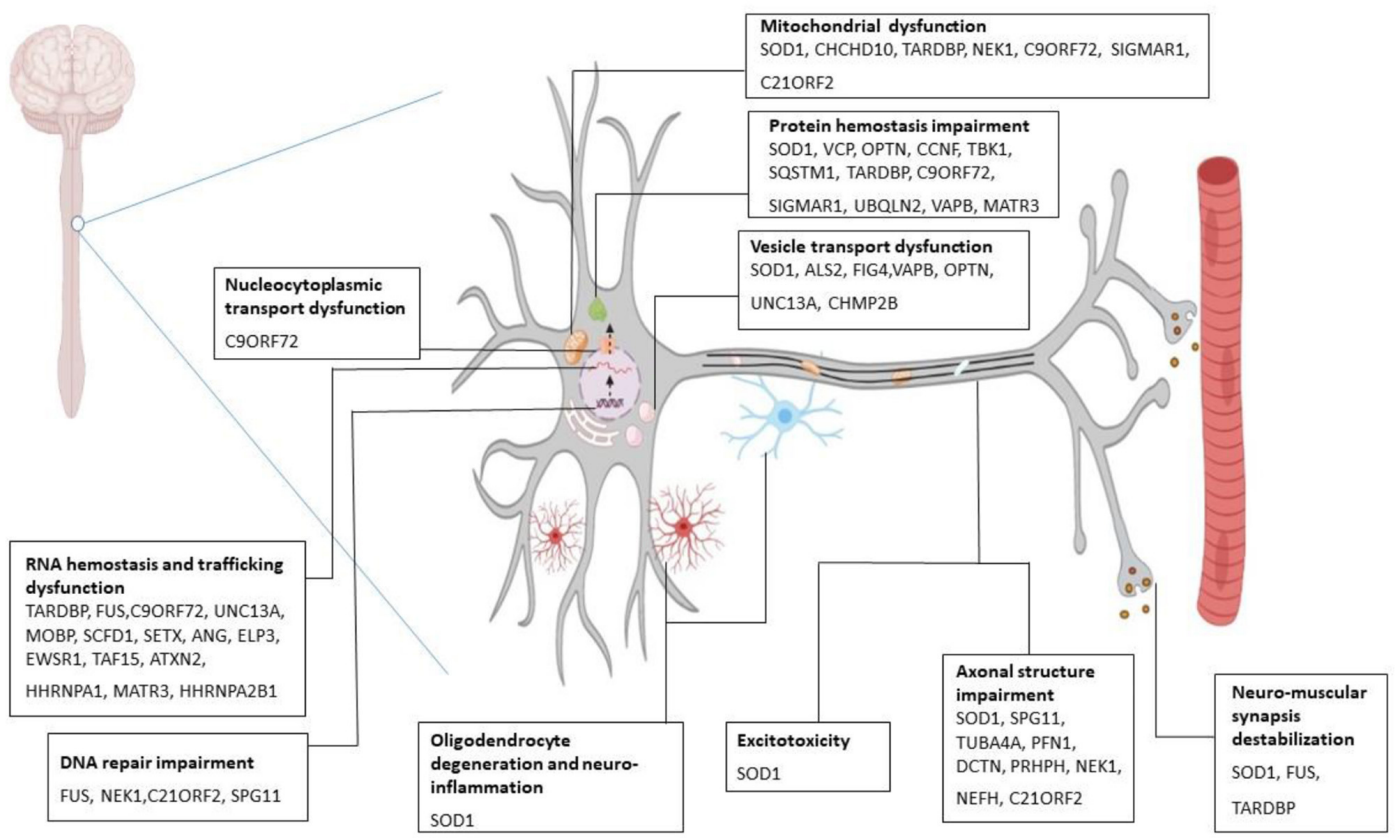
Molecular mechanism of ALS pathogenesis. Several gene mutations are discovered to be associated with amyotrophic lateral sclerosis (ALS). Mutant SOD1 leads to cytoplasmic inclusions, chaperone dysregulation, and reduction in components of ubiquitin-proteasome system, oxidative stress, incorrect protein imports, and mitochondrial dysfunction. Mutant C9ORF79 causes the formation of RNA foci that can sequester RNA binding proteins and impair the translational process. Also, it produces dipeptide repeat proteins and causes neurotoxicity. Mutation in TARDBP (the gene which encodes TDP-43) gives rise to toxic aggregation of TDP-43 and impairs endosomal trafficking and mitochondrial function. FUS mutation perturbs DNA repair, RNA metabolism, and synaptic function (21–30). SOD1, superoxide dismutase; FUS, fused in sarcoma; TARDBP, transactive response DNA binding protein 43.

Pathologically, ALS is associated with processes including, protein hemostasis, aberrant intracellular and vesicular transport, impaired RNA metabolism, deviant DNA repair, excitotoxicity, and mitochondrial dysfunction which leads to clinical manifestations of the disease (Figure 1) (21–30). Therefore, the diagnosis of ALS is based on family history, clinical presentations, and a series of tests (31). However, in the early stages of the disease, accurate and early diagnosis can often be difficult due to some identical early symptoms between ALS and some other neurological or neuromuscular disorders. Additionally, in ALS conditions, neither two individuals may demonstrate similar phenotypes from the onset of symptoms to disease progression. Accordingly, errors or delays in ALS diagnosis can be problematic for rapid treatment, which can finally lead to considerable effects on patients' survival rates (32). In ALS treatment, some pharmacological (e.g., Riluzole, Edaravone) (33) and non-pharmacological approaches (e.g., special diets, nutritional supplements, acupuncture) are current, which can slow down the progression of the disease and relieve symptoms. However, there is no convincing evidence of a definite treatment to reverse the effects of ALS (34). The problems of preclinical and clinical stages involved in drug discovery and development platforms are a number of the main reasons that make the ALS treatment process face many challenges. Different factors such as the poor correlation of preclinical pharmacokinetic assessments with in-clinical results, the lack of appropriate animal models, phenotypic dissimilarity of the same mutations, and costly experiments to check the ALS genetics are among the problems in preclinical and clinical stages, which can have a major role in drug discovery and development failure (35). Thus, it is time for new advanced bioengineering technologies to step into the arena to provide a suitable platform for studying the complexities of disorders and examining the effects of various drugs.

With the advancement of bioengineering technologies, novel techniques and systems such as micro-physiological systems have emerged as revolutionary leading-edge technologies in biological and medical sciences. Organ on a chip (OOC) as an innovative bioengineering tool aims to provide a platform to promote new studies for discovering novel therapeutic approaches for different disorders (36). In ALS conditions, OOC not only helps to elucidate the mechanisms underlying the pathophysiology involved in ALS but also can be an effective step in the assessment and screening of new candidate drugs in drug discovery and development (37). To this end, interdisciplinary collaborations and applying novel strategies such as stem cell and microfluidic technologies contribute to accomplishing the OOC platform and enhancing the performance of this microelectromechanical system (38).

Therefore, this review initially focuses on an underlying knowledge of ALS pathogenesis, the current therapeutic management of ALS patients, and common *in vitro* and *in vivo* modeling platforms that are applied in the drug discovery and development of ALS. In particular, then the OOC as an innovative biomimetic platform in disease modeling is introduced. Finally, the studies on ALS and pharmacological approaches examined on the chip platform are described.

## Clinical Hallmarks of ALS

**Figure 2:**
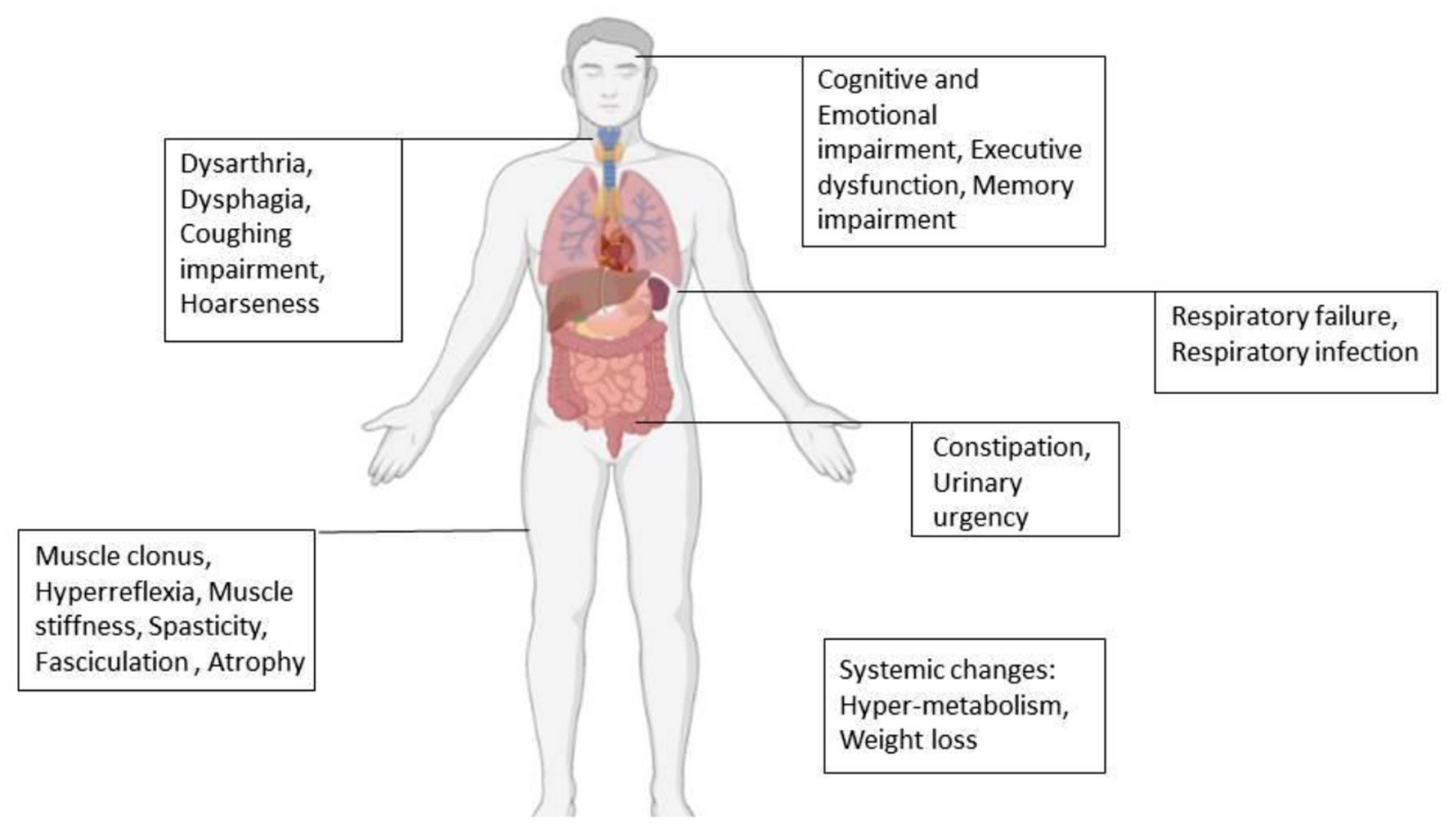
Motor, non-motor, and systemic changes in ALS (21, 43).

Amyotrophic lateral sclerosis (ALS) is a progressive disorder characterized mainly by painless paralysis, which begins focally but then spreads to most muscles of the body. Finally, the disease leads to death within 3–5 years, mostly due to respiratory failure caused by diaphragm paralysis (39, 40). Based on the type of motor neuron involved, clinical presentation differs. Loss of UMNs results in clonus, hyperreflexia, muscle stiffness, and spasticity. Loss of LMNs initially presents with fasciculation muscle cramp due to electrical irritability and in final stages presents with muscle atrophy (25, 39–42). Besides motor changes, a wide range of non-motor signs and symptoms can be seen in ALS (Figure 2) (21, 43).

## Current Therapeutic Approaches and Managements

ALS is a progressive disease that has a high rate of morbidity and mortality. Although up to now there is no definite cure for the disease, multiple medical therapies have been proposed and passed the trials successfully to maximize the quality of life and minimize morbidity (44, 45).

One approach in this regard is symptomatic management. Currently, the most common treatment for cramps is quinine. Besides this treatment, physiotherapy and hydrotherapy may be helpful. However, Levetiracetam is a treatment of the first step recommended by European guidelines (44, 46). Another physical complaint in ALS is spasticity. To address this problem, physiotherapy and exercise therapy like passive stretching and postural exercises combined with pharmacological treatment (like oral muscle relaxants such as Baclofen and Dantrolene and intrathecal Baclofen) have shown to be helpful. Currently, it has been suggested that Botulinum toxin A can also be a part of treatment (44, 46, 47). One more physical manifestation of ALS is pain. A combination of physiotherapy, rehabilitation, and pharmacotherapy is the key to pain management. All classes of analgesics can be used based on the etiology of pain, with special consideration of opioids as the analgesic of choice (44, 46).

As mentioned previously, ALS patients may suffer from psychological symptoms like Pseudobulbar affect as well. SSRIs and TCAs are the most common therapy for this problem. Recently, a combination of dextromethorphan and quinidine is also recommended (44, 46, 47). Other mood disorders like depression and anxiety can be treated with psychiatric interventions and benzodiazepines. Also, Modafinil may be useful for fatigue reduction (44, 46). In order to deal with dysphagia, ALS patients should consume a high-calorie and high-protein diet as well as oral nutritional supplements. Proper posturing while eating prevents aspiration. Regular nutritional assessment is recommended and if the patient suffers from advanced dysphagia, enteral nutrition will be helpful (39, 44, 46–48).

Respiratory failure is the main cause of mortality and morbidity in ALS. This happens due to diaphragm weakness, inefficient cough, and respiratory infection (44, 46, 47). To address these challenges different strategies have been proposed. Non-invasive ventilation (NIV) is the method to support ventilation with intermittent positive pressure given to patients with a facial or nasal mask. Another method is invasive mechanical ventilation (IMV) which contains bypassing the upper airways by inserting tracheostomy or endotracheal tube. This method also increases survival. To manage the impaired secretion clearance, anticholinergics are commonly prescribed, while taking into account their effect in drying the mouth and thickening the secretions. Intraglandular botulinum toxin, radiotherapy, mucolytics, and respiratory humidification are other therapies that may have beneficial effects in secretion management (44, 46–49).

Besides symptomatic management, up to date, there are 2 more medications that have the hope to help ALS patients. Riluzole is an antiglutamatergic substance that decreases excitotoxicity and has been shown to increase survival up to almost 3 months more, especially if applied in the early stages of the disease. However, according to the researches, it doesn't affect muscle strength significantly and has adverse effects like hepatic failure and pancreatitis. Edaravone is an antioxidant agent that reduces lipid peroxides and hydroxyl radicals. Nevertheless, how exactly the agent improves ALS and who the target population is for the drug, are the questions that need further investigations to be answered (39, 45). In addition, there are some therapeutic strategies that are at the research level (50). As an example, research has shown that Rasagiline is an anti-apoptotic agent which has a positive effect in the treatment of ALS especially if combined with riluzole. Rasagiline maintains mitochondrial membrane potential and reduces oxidative stress and improves motor functions (26). Also, according to research, administering neurotrophic or neuroprotective agents like 7,8-dihydroxyflavone or glycoprotein non-metastatic protein B can promote neuronal regrowth and neuronal repair (39, 45). Furthermore, some studies have focused on the role of cell-based therapies for neurodegenerative diseases (51–53).

Astrocytes are known to have a crucial role in the metabolism and homeostasis of neurons and maintaining the micro-architecture of the nervous system. Thus, transplantation of astrocytes and glial-restricted precursors (which later differentiate to astrocytes) can stimulate neural regeneration. Also, neural stem cells (NSCs) are multipotential progenitors that have the ability to self-renewal and differentiation into neural cells. In the condition of neurodegeneration, these cells promote neurogenesis in the spinal cord. However, the amount of indigenous NSCs is not enough to prevent the progression of the disease. Hence, transplanting NSCs to ALS patients can strengthen the process of neurogenesis and angiogenesis as they secret neurotrophic agents and have anti-inflammatory and immunomodulatory roles (26, 45). In conclusion, although multiple therapeutic approaches have been proposed for ALS up to now, the lack of a definite promising treatment compels further investigation in this field.

## ALS *in vivo* Modeling Platforms

In order to achieve a better understanding of ALS to unravel its complexity and discover new therapies, a wide range of models have been used by researchers (54). As an example, budding yeast Saccharomyces cerevisiae is a eukaryote unicellular model for ALS. The genome of this model is completely sequenced and has orthologs for many human genes. RNA-binding protein ATXN2 was first discovered in Saccharomyces cerevisiae as a modifier and interactor of TAR-DNA binding protein 43 (TDP-43) and a CAG trinucleotide expansion in this gene causes it to be a risk factor for ALS. Moreover, it was figured out that GGGGCC repeat expansions in C9orf72 are associated with nucleocytoplasmic transport dysfunction and neurotoxicity (55, 56). It is also reported that superoxide dismutase (SOD-1) mutation in yeast leads to protein instability, metabolic regulation perturbance, and cell senescence (57). Fused in sarcoma (FUS) overexpression inhibits the ubiquitin-proteasome machinery and causes toxic cytoplasmic aggregations. Additionally, it has been discovered that high expression of TDP-43 has a cytotoxic effect by forming aggregations that can inhibit cell growth.

Drosophila melanogaster has presented a valuable platform to study ALS disease. This model has a short generation time. Its genome is fully sequenced and its nervous system is sophisticated and contains 100,000 neurons and can manifest some of the ALS phenotypes. Besides, a powerful genetic toolbox has been developed to manipulate genes and neuronal functions of this fly (55, 56, 58–60). Drosophila expressing mutant human SOD1 (G85R, A4V, G37R, G41D) presents motor dysfunction (climbing impairment), SOD1 aggregation, reduced synaptic transmission, oxidative stress, mitochondrial dysfunction, and reduced life span (55, 58). This model has shown that TDP-43 and FUS are essential proteins for neurons and any overexpression or deficiency in the genes encoding them is toxic for neuronal health (56). Mutant human FUS expressed in transgenic flies has been reported to perturb nucleocytoplasmic transport, transcription and translation regulation, Hippo-signaling pathways, stress granule assembly, and miRNA biogenesis (55, 58). On the other hand, upregulation and overexpression of wild-type or mutant human TDP-43 lead to cytoplasmic TDP-43 aggregation, morphological change in the neuromuscular junction, and cell death. Thus, they result in reduced life span, axonal transport impairment, eye degeneration, and loss of motility (55, 58). Likewise, this model demonstrated that muted C9ORF72 causes ommatidia, motor defects, and neuromuscular junction abnormalities, and its pathogenesis can be affected by the translational process, nucleocytoplasmic transport, and protein formation (55, 56, 58, 59, 61).

The nematode *Caenorhabditis elegans* is another model accepted for ALS studies. It has a short life cycle of 3.5 days. The worm is transparent; thus proteins can be monitored in an *in vivo* condition. Its nervous system is simple and well-characterized which contains 302 neurons and synapses which use mammalian neurotransmitters. Almost 30% of human genes have orthologs in *C. elegans* and its genetic material can be manipulated easily due to a rich toolbox available for genetic studies in this worm (55, 56, 58). Through these studies, it is revealed that ALS pathogenesis contains both loss-of-function and gain-of-toxic-function mechanisms. It has been reported that SOD1 mutation in this model prevents natural protective responses to oxidative stress and causes protein aggregation. Hence, the mutation leads to locomotion defects and neuronal transmission impairment (55, 58). Also, transgenic expression of mutant FUS shows mislocalized cytoplasmic aggregation of this protein and is associated with reduced transmission of synaptic vesicles from neurons to muscles at the neuromuscular junction (55, 58, 62). Moreover, expression of mutant TDP-43 in GABAergic motor neurons causes neurodegeneration, synapses impairment, progressive age-dependent motor defect, and increased endoplasmic reticulum (ER) stress (55, 58). On the other hand, it has been shown that mutation in alfa-1 (ortholog of C9ORF72 in the worm) is linked to neurodegeneration (especially in GABAergic motor neurons), motility defect, and vulnerability to osmotic stress (55, 58).

Danio rerio is a powerful vertebrate model to study neurodegenerative diseases (63). Its advantages include transparency of embryos, easy-imaging, available behavioral tests to examine motor activity, short life cycle, and high fertility. Its easily modifiable genome is highly similar to the human genome and conserves the genes responsible for neurodegenerative diseases (55, 56, 58). Overexpression of mutant SOD1 in zebrafish manifests motor neuron and muscle degeneration, neuromuscular junction abnormality, locomotor impairment (especially swimming), and reduction in life span. Also, it causes interneuron dysfunction leading to reduced inhibitory messages to motor neurons of the spine (55, 56, 58). On the other hand, studies in tardbp mutations discovered an association between these mutations and motor neuron axonopathy, reduced acetylcholinesterase expression, and hyperbranched ventral root projections (55, 58). Mutant human FUS in zebrafish results in impaired nuclear import, accumulation of stress granules, and locomotion defects. Also, it causes ventral root projection abnormalities, reduction in synaptic transmission, and abnormal synaptic structure. Furthermore, a mutation in c13h9orf72 which is an ortholog of *C9ORF72* invokes impaired locomotion and abnormal ventral root projections (55, 56, 58).

Rodent models including mice and rats are the gold standard of preclinical studies of neurodegenerative diseases due to their physiological and genetic similarities to those of humans. They have large brains and body sizes and can be easily manipulated due to their stress resistance (55, 56). Hence, these models have been widely used in modeling genetic mutations of ALS. These models have shown that misfolded SOD1 is secreted from motor neurons and can spread to the neighboring cells in a prion-like manner. Mutant SOD1 rodents have boosted our understanding of the role of neuroinflammatory and non-neural cells like glial cells in the pathology of the disease. These mice demonstrated that alterations in mitochondrial functions and structure brings up energy failure and oxidative stress. Also, they revealed that ALS is indeed an axonopathy caused by changes in cytoskeletal organization and transport of RNA and vesicles which leads to axonal degeneration and retraction from muscles (56). Rodent models that underwent FUS mutation display paralysis, gliosis, neurodegeneration, neuromuscular junction abnormality, protein aggregation, loss of transportation between Golgi complex and endoplasmic reticulum as pathologic features of ALS. Furthermore, in transgenic TDP-43 mice, previous pathologies, as well as DNA damage, are observed. Mutation and mislocalization of TDP-43 inhibit DNA repair, leading to DNA damage as an additional pathological feature of ALS. Likewise, C9ORF72 mutation in mice is reported to be associated with locomotion defect, weight loss, myopathy, cognitive, and behavioral changes like anxiety and hyperactivity (55, 64).

Animal models are used widely since a long time ago to validate the preclinical results achieved by *in vitro* studies and to prepare them to be used in human beings. However, these models are expensive and bring up ethical matters (36). Also, some the animal models such as rodents require much time to generate and be investigated (55, 56). Lack of complexity and anatomical difference with humans are other obstacles seen while working with animal models. As an example, Saccharomyces cerevisiae is a unicellular model and is unable to show complex phenotypes of ALS and advanced cross-talks between motor neurons and their environmental cells. The nervous systems of Drosophila melanogaster and *Caenorhabditis elegans* are anatomically different from that of human beings and the CNS of zebrafish lacks upper motor neurons (55, 56, 65). On the other hand, the immune system of animals has shown significantly different responses when being exposed to new pharmaceutical agents (36). Animal models are not suitable to test multiple simultaneous hypotheses, as well. Moreover, most of the animal models discussed here are overexpression models. Thus, considering all these limitations as well as the need to test knock-in hypotheses imposes genetic engineering to develop novel platforms like OOC by using stem cells (55, 56).

## ALS *in vitro* Modeling Platforms

Despite all the discoveries made in the field of ALS, yet our understanding of the disease progresses slowly due to the lack of an appropriate model. Animal models have been used widely to study neurodegenerative diseases; however, not all of the mutations related to fALS can be examined in animals (66). Meanwhile, they are not good candidates to investigate sALS cases who are almost 90% of patients. Moreover, drugs developed in these models have shown to be ineffective in humans due to interspecies differences, considering the difference between the human central nervous system and the system in animal models as an example. Thus, to improve our understanding, the establishment of patient-specific models, using regenerative medicine is crucial (29, 67).

Stem cells have great potential to proliferate and differentiate into various cells including those that are difficult to isolate from patients (68, 69). One type of stem cell which can be used for this purpose is human embryonic stem cell (hESC) (29). They are known to be valuable tools for SOD1 mutation studies and have shown that cells with this mutation present mutation-dependent reduction in axonal length (70). However, the process of elaborating motor neurons from hESCs is a time-consuming one (almost 2 months) in which 80% of the final cells maintain undetermined identity and only 20% of them are motor neurons (MNs). Besides, not all the genetic mutations can be represented in these cells. Hence, they are not frequently used in ALS studies (29, 70). Another novel model for neurodegenerative disease is induced pluripotent stem cells (iPSCs), pluripotent cells with the ability of self-renewal like ESCs. These cells are elaborated by genetic reprogramming of skin fibroblast and peripheral blood mononuclear cells and reverting them to their ESC-like state. Therefore, they are more available in contrast to ESCs which could be acquired mainly from embryos after *in vitro* fertilization (67). Moreover, using patient-derived iPSCs provides the opportunity for researchers to study the disease mechanisms in the same way as it happens in the patient body (29, 67, 70).

Stem cell modeling of ALS has shown that A4V mutation of SOD1 causes a proapoptotic phenotype, reduction in neurite outgrowth, and survival while E100G mutation leads to activation of endoplasmic reticulum stress pathway, extracellular signal-regulated kinase (ERK), and c-Jun N-terminal kinases (JNK) signaling pathways (28, 29). On the other hand, iPSC-derived motor neurons with A4V or D90A mutations in SOD1 exhibited neither protein aggregation nor mitochondrial abnormalities, implicating the less importance of these pathologies in human ALS compared with animal models. However, neurofilament aggregation is documented to be an early mechanism involved in ALS which leads to axonal pathology and neurite swelling and is a possible therapeutic target for ALS (28, 29, 71, 72). Also, SOD1A4V mutation has proved to cause a reduction in the activity of delayed-rectifier potassium currents in motor neurons and subsequently, causes hyperexcitability. Thus, blocking hyperexcitability by using retigabine has been shown to increase the survival of motor neurons (28, 29, 56, 72). Finally, mutations in SOD1 give rise to transcriptional abnormalities in genes associated with mitochondrial dysfunction, increased oxidative stress, intracellular transport impairment, and activation of ER stress pathways (29).

In an iPSC modeling study, Q343R, M337V, and G298S mutations in TDP-43 were associated with the decreased protein of neurofilament RNAs, increased TDP-43 RNA, and accumulation of TDP-43 with cytoplasmic granules (28, 71–73). Other studies displayed that iPSC-derived MNs with S939L and G294V mutation in TDP-43 exhibited axonal transport dysfunction, neurofilament accumulation, and survival reduction. They also suggested that upregulation of TDP-43 and loss of nuclear TDP-43 is the key pathology of the disease and granule accumulation may be seen only in delayed stages of ALS (28, 72). Moreover, according to studies, exposure to stress in iPSC-derived MNs provokes the formation of cytoplasmic TDP-43 liquid droplets that later, causes nucleo-cytoplasmic transport dysfunction, nuclear TDP-43 depletion, and cell death (29). One more study offers the possibility that reduced synaptic activities at early stages of ALS are caused by a decrease in voltage-activated Na+ and K+ currents which results in neurodegeneration (29, 74). On the other hand, TDP-43 depletion is found to reduce the expression of neuronal growth-associated factor stathmin-2 which later, inhibits neurite regeneration (29).

Recent studies using iPSC-derived motor neurons harboring C9orf72 mutation exhibited a toxic gain of function of RNA in ALS. The repeat expansion of the gene leads to the formation of RNA foci which sequester RNA binding proteins hnRNAP1 and Pur-α as well as ADARB2. These foci impair the translation of mRNAs and cause excitotoxicity (28, 29). Also, the same studies reported that GGGCC repeat expansion leads to toxic protein formation like dipeptide repeat protein species (DPRs). DPRs can cause neurotoxicity through different mechanisms. DPRs are found to cause mitochondrial dysfunction, oxidative stress, and DNA damage. Moreover, they may disrupt the pre-mRNA splicing and decrease the translational activity of MNs as well (29, 75, 76). Furthermore, in a study by Selvaraj et al., MNs from iPSCs with C9orf72 mutation demonstrated the upregulation of GluA1 AMPAR subunits. This matter leads to Ca^2+^ homeostasis dysfunction and vulnerability to excitotoxicity (28, 29, 75, 77). One more piece of data gained by the study of C9orf72 mutation is that in the early stages of ALS, MNs undergo a hyperexcitability state but later, manifest hypo excitability (29, 75). Another data gained by the study of C9orf72 mutation is that in the early stages of ALS, MNs undergo a hyperexcitability state but later, manifest hypo excitability. This change of excitability may be due to a decline in voltage-activated Na+ and K+ currents, implicating the role of ion channel dysfunction, and electrophysiological activity alteration in the pathogenesis of ALS (29, 75). Finally, C9orf72 mutation is discovered to be associated with dysfunction in nucleo-cytoplasmic trafficking of RNA binding proteins like ADAR2. This causes mislocalization of RNA binding proteins and as a consequence, aberrations in the RNA editing process of the genes related to transcription (28, 29). Using iPSC-derived MNs for studying FUS mutations, other aspects of the disease pathology were elucidated. Mutations in the FUS nuclear localization sequence was found to impair DNA damage response (DDR) signaling, leading to mislocalization, and aggregation of FUS in cytoplasmic space in a reciprocal way: DNA damage causes FUS mislocalization and impaired FUS shuttling to cytoplasm provokes DNA damage, giving rise to neurodegeneration in the ultimate step (28, 29). In addition, miRNA dysregulation is seen in mutant FUS MNs to be caused by aberrantly upregulated RNA-binding proteins and apoptotic factors (29). In the same way, it has been demonstrated that FUS mutation is connected to mitochondrial and ER vesicle transportation defect, axonal degradation, and mitochondrial structural disruption (28, 29).

Conventionally, stem cells were cultured on a rigid platform with a composition like ECM to promote cell adhesion, proliferation, and differentiation. The 2D models obtained by this method were cheap and reproducible; however, they lack the complexity and 3D organization of body organs. They are not able to recapitulate the cell-environment and cell-cell cross talks as they happen in the *in vivo* condition and subsequently, fail to mimic the exact mechanisms of diseases. Hence, they were substituted by 3D models (78). In order to develop 3D cultures, stem cells including iPSCs are grown in synthetic or natural scaffolds that perform as ECM or they are settled to self-assembly and produce the ECM by themselves. They can provide complex spatial structures that are relatively appropriate to manifest biological interactions (78). With the aim to study ALS disease, 3D models are developed in which neuron-glia interactions and NMJ are displayed. These models can be used to study changes in NMJ as the main cause of disease signs and symptoms. Also, they are used to explore disease mechanisms and probable therapeutic strategies (72). Despite all the fruitful discoveries made by using these models, 3D cultures are expensive and need many experts to handle the challenges of developing them like reproducibility, scaffold materials and components, and their homogeneous distribution. Before using them in clinical investigations, the matter of safety in different stages like dedifferentiation of cells and immune reactions, as well as ethical and legal matters, should be ascertained. Lack of optimized protocols to elaborate these models and risk of contamination with unwanted cells are other challenges that need to be addressed. Since these models are difficult to image and microscopy, along with the other obstacles, implicate the need for further progress and optimization in the extension of this technology (78–84).

## OOC as a Potential Strategy for Disease Modeling and Drug Discovery

To date, many significant advances have been made using some *in vitro* and *in vivo* models to fill gaps in the current knowledge of neuroscience. However, they still cannot properly demonstrate the physiological complexities of the human nervous system. Hence, these constraints highlight the need to seek efficient modeling methods instead of previous ones (85, 86). Accordingly, multidisciplinary efforts were made to develop an appropriate modeling method that can mimic the biology of the human body systems with an eye toward different therapeutic purposes. As a result of these studies, OOC was proposed as an alternative biomimetic approach, allowing further advances in disease modeling, drug discovery, and personalized medicine (87).

Organ on a chip (OOC) is considered a micro-physiological device, which has recently taken the front seat in bringing a new perspective to the study of the mechanisms underlying biological systems in an *in vitro* environment almost the same as that of humans (88). Structurally, OOC is a small chip made of flexible polymers (89), which consists of four interacting components, including cells, microfluidics, stimulus components, and sensors that form a biological platform and are used for biomedical and pharmaceutical studies (88). ESCs are one of the cellular sources candidates used in OOC technology (90). Additionally, the past decade has witnessed important advances in patient-derived iPSCs technology, which have made significant opportunities in the progress of different branches of biomedical science (91, 92). Recently, in light of rapid progress in developing microfluidic cell culture devices, remarkable efforts have been achieved in employing iPSCs, as a pioneering approach in OOC technology to recreate the biological systems in a 3D environment. To produce iPSCs cell lines, the patient's mature and specialized cells are reverted into states of pluripotency through the cell reprogramming process. Consequently, generated iPSCs can be propagated and manipulated to differentiate into various cell types depending on the purposes. Therefore, iPSCs can create significant opportunities to imitate the physiological and pathophysiological mechanisms of the patients by providing an unlimited source of any human cell type possessing the patient genetic background. Therefore, it can be concluded that the integration of OOC technology with the iPSC strategy can be an efficient step in discovering new therapeutic approaches and exalting personalized medicine by developing human tissue equivalents (89).

In parallel with living cell tissues, microfluidics is also considered an advanced technology used in micro-physiological chips, which is responsible for supplying, controlling, regulating, and making changes in fluidic flow by confining them in tiny multi-channels with functional dimensions in the micrometer range (93). Indeed, the main advantages of microfluidics can be studied in four categories. Firstly, controlling the cell patterning process is of great importance that microfluidic technology is responsible for it within OOC to provide specific functionality and complexity of a living organism's physiological conditions (88). Cell patterning refers to a process in which cultured cells in high density and with different phenotypic characteristics are arranged in a precise position on an *in vitro* platform in a controlled manner. To create such an arrangement of cells, various methods such as electrical and optical guiding, direct inkjet cell printing, and laser-assisted bioprinting (LAB) technologies are applied in tissue engineering (94). Secondly, microfluidics can provide the frictional force of a biological fluid flow (referred to as shear stress) by different perfusion pump systems to supply the materials needed by the cells and remove the excretory materials (88). *In vivo*, shear stress is mainly due to the functioning of the body's vascular system (95), which can lead to alterations and regulation in various biological processes (e.g., gene expression, signaling pathways, differentiation, proliferation, and migration) (96). Thirdly, since microfluidic technology is a manifestation of the extracellular environment, it can create a concentration gradient of biochemical molecules by various techniques, such as changes in flow rate or channel diameter. Subsequently, cellular signaling mechanisms, under the influence of this concentration gradient can affect the cellular biochemical processes. And finally, loading of the necessary mechanical stresses through the porous membranes to the cells is one of the advantages of microfluidic technology, which occurs naturally under physiological conditions due to the pressures mediated by some of the tissues within the human body (e.g., bone and lung) (88).

As mentioned in features related to microfluidic technology, it can be understood that the presence of various mechanical stimuli has a significant contribution to mimicking the complexity of native tissues. Regarding biomechanical stimuli, it can be stated more precisely that the effect of mechanical forces in the regulation of cellular and tissular functions is possible through the mechanotransduction process. In other words, immediately after receiving mechanical forces, the cells convert them to cellular signals. Consequently, appropriate biological responses can be elicited at the earliest possible time against physical forces, which ultimately lead to dynamic reciprocity between cells and the surrounding environment within a tissue or an organ (97). In addition, it has gradually become clear that mechanical forces not only play a role in establishing a biologically stable system but can lead to the progression of various diseases such as cancer in case of disruption. Therefore, depending on the type of engineered tissue and the purpose of the study, various physical stimuli (e.g., shear flow, compression, and stretch/strain) can be exerted on the cells through microfluidic technology, which demonstrates superiority over other *in vitro* modeling methods (98). In addition to mechanical stress, biochemical, and electrical stimulations are involved in mimicking biological conditions provided to the cells cultured (99). Regarding biochemical stimuli, vascular endothelial growth factor (VEGF) is one of the common examples which has a crucial role in stimulating endothelial cells to promote the formation of new blood vessels. In addition, Angiopoietin 1 (ANG-1) as another biochemical factor, can modulate the activity of the endothelial cell-specific receptors (like Tie2) to maintain endothelial cells and construct vascular branching. Moreover, bone morphogenetic protein 4 (BMP4), fibroblast growth factor (FGF), Sonic hedgehog (SHH), and retinoic acid (RA) can be categorized as other types of biochemical stimuli which can adjust the differentiation of stem cells and their development into neural sub-types. In another group, Brain-derived neurotrophic factor (BDNF), Neurotrophin-3 (NT-3), and Nerve growth factor (NGF) are involved in maintaining and controlling the survival, function, and plasticity of neurons. Therefore, considering the basic role of the mentioned biochemical factors, it is concluded that regulating the concentration gradient of biochemical stimuli in microfluidic chips is one of the most important points that can increase the similarity of the *in vitro* platforms with the *in vivo* environments (100). In addition to biomechanical and biochemical stimuli, electrical impulses also play a key role in the micro-physiological systems. A well-known example of electrical stimuli applications is the simulation of heart cells functions by applying electrodes in the microfluidic chips. In the end, it should nonetheless be noted that the combination of all three stimuli can be a major step toward achieving close imitation of the human body system (99).

Obviously, the increased complexity of OOC in structure and function due to the integration of the basic components highlights the need for a monitoring system to measure the chemical and biological parameters during the disease modeling and drug screening process. To overcome this problem, the use of exquisitely sensitive sensors, as an *in situ* monitoring method, has been developed to assist in scrutinizing the biological mechanisms by analyzing the target substances and parameters which are related to the physiological and pathophysiological conditions under the study. In addition, the automatic operation of sensors can eliminate the need to hire many research technicians in longitudinal studies or the projects that are accompanied by conducting a multitude of chemical, biological, or pharmaceutical analyses (101). Since a wide variety of biological and chemical parameters are involved in the functions of a living system, various types of sensors come in many forms to interact with the OOC performance including, but not limited to mechanical, impedance, electrochemical, and biomarker specific sensors that have been assigned to evaluate stimulation, cell behavior, pharmacological and toxic effects of drugs, environmental parameters, metabolic activity, etc. (101, 102).

As demonstrated above, the final performance of the chips is achieved by synchronizing the various components in the micro-physiological system. Hence, it can be concluded that creating a constructive interaction between all of these components can pave the way for the creation of a biological system that mimics the human body's condition. As a result, a fundamental step can be taken toward modeling diseases and discovering various drugs with higher therapeutic potential, which can be along the lines of reducing the use of *in vivo* models and increasing productivity accompanied by minimizing cost and time used in drug discovery and development pipeline (89).

## ALS Disease Modeling On-A-Chip

**Figure 3:**
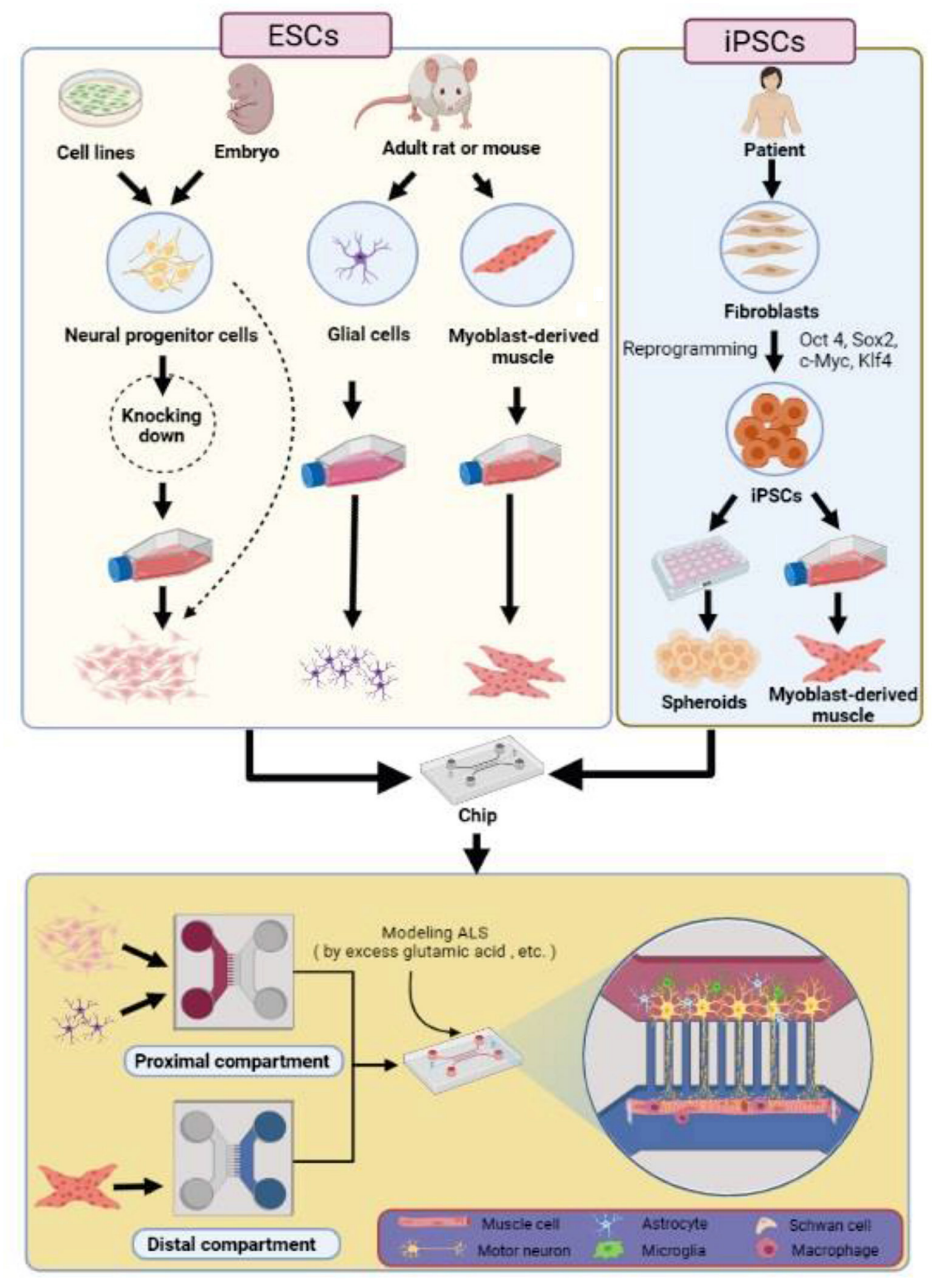
ALS modeling on a chip. To create the ALS model on a chip, two cell sources can be used, which include ESC and the patient's somatic cells. In the case of ESCs, neural progenitor cells can be obtained from cell lines and mice or rat embryos. Neural progenitor cells can be used directly in the chip or after cell culture. In addition, neural progenitor cells can be combined with optogenetics technology to express light-sensitive channelrhodopsin-2. To obtain muscle and glial cells, adult mice can be applied. Both Muscle and glia cells are used in the chip after culturing. In the case of using iPSCs, somatic cells must be forced to express exogenous transcription factors (Oct4, Sox2, Klf4, and c-Myc) through the reprogramming process. iPSCs can form different types of human cells, such as skeletal muscle myoblasts and NSCs. Skeletal muscle myoblasts create mature skeletal muscle cells. NSCs create nervous system cells. To create an ALS model, skeletal myoblast cells are first embedded with a collagen gel into the distal compartment of the chip. The muscle-engineered cells begin to grow and differentiate while they hover between two pillars. NSCs are then embedded in the proximal chamber of the chip along with the collagen gel. NSCs express a series of factors that form motor neurons and elongate axons toward muscle cells. In addition to astrocytes, cells such as microglia, Schwann cells, and macrophages should be included in the chip. As the cells mature, the structure of the NMJ completes. Some techniques such as adding high concentrations of glutamic acid to the culture medium are used to demonstrate the NMJ dysfunctionality to model the ALS disease. Techniques such as time-lapse microscopy, calcium imaging, pillar displacement, partied image velocimetry, image subtraction video recording, mitochondrial dyes, axon-seq, FISH, and immunocytochemistry applied to evaluate the NMJ model within microfluidic chips (75, 90, 112, 113). ESCs, Embryonic stem cells; FISH, fluorescence *in situ* hybridization; GFAP, glial fibrillary acidic protein; iPSCs, induced pluripotent stem cells; NMJ, Neuromuscular junction; NSCs, Neural stem cells; PDMS, Polydimethylsiloxane.

Clinically, in ALS conditions, degeneration of UMNs or LMNs is considered a phenomenon involved in NMJ dysfunctionality (103). Biologically, the NMJ function is mediated through a tripartite architecture between presynaptic motor neuron terminals, postsynaptic muscle fibers, and synapse-associated glial cells (104). Additionally, in terms of function, NMJ is responsible for transmitting electrical impulses from the nervous system to the muscle fibers through motor neurons. Considering critical NMJ function, any disruption in this cellular junction can have devastating effects on the individual, which is precisely observed in the pathogenesis of ALS (105). Over the past years, several studies have been conducted on NMJ studies by using homologous [e.g., human-human (106) and rodent-rodent cells (107)] and heterologous (e.g., human-rodent) co-culture models on laboratory platforms (108–110). Therefore, traditional cell culture platforms (e.g., Petri dishes and multi-well plates) are among the common *in vitro* approaches intending to replicate *in vivo* biological mechanisms over the past years. However, the cell-cell, cell-ECM interactions, and the role of intricate signaling networks are not adequately mimicked by mentioned *in vitro* platforms. On the other hand, some of the methods which can increase the similarity between *in vitro* platforms and *in vivo* counterparts are also high-priced and/or require considerable effort and time (111). Over the recent years, the emergence of microfluidics and OOC technology, as the groundbreaking advances in the field of biomedical sciences, could provide opportunities to facilitate translational researches from bench to bedside in various neurodegenerative disorders, especially ALS (100). In OOC technology, the pathophysiological mechanisms of ALS and the effects of drugs have been investigated by focusing on NMJ modeling (Figure 3) (114). Drawing on a range of stem cell sources, scientists created various NMJ-on-a-chip structures. Rodent myoblast-derived muscle cells and ESCs-derived motor nerve cells are groups of cell types, which are employed in microfluidic devices using compartmentalization. For instance, Southam et al. developed a neuron–neuromuscular junction model on a microfluidic device via culturing the embryonic stage neonatal rat-derived motor nerve cells, spinal cord-derived glia cells, and myotube-derived myoblasts. In this study, motor nerve cells of embryonic rats were initially embedded in one chamber containing poly-l-laminin. While glia and myoblast cells culturing was first performed in separate flasks (poly-l-lysine-coated flasks for glia cells and collagen-coated flasks for myocytes) and then embedded in the special chambers of the chips on 2 days and 2 weeks after nerve cells embedding, respectively. In this study, special attention has been paid to the role of non-neuronal cells along with motor neurons. Examination of the cells in the chip chambers indicates that cultured glia cells include astrocytes, oligodendrocytes, microglia, and a small number of fibroblasts. Although glial cells play a key role in the development of motor neurons by making contact with motor neurons and supplying neurotrophins, the survival of motor nerve cells largely depends on the formation of NMJ. In this regard, the formation of acetylcholine receptors has been suggested as one of the confirmation strategies for the formation of NMJ in micro-physiological systems (115).

In another study, the investigation of the NMJ formation and functionality was carried out by Ionescu et al., in which the mouse myoblasts originating in gastrocnemius muscle satellite progenitor cells and the Hb9::GFP labeled motor neurons have been cultured in compartmental chambers. The noteworthy point in this study is that applying different techniques such as fluorescent staining, live imaging of muscle contractions and the measurement of intracellular Ca2+ could open new doors into the biology of motor neurons and muscle communications in this model. In addition, this study highlights the importance of accurate insight into the possibility of testing different drugs in the NMJ model for scientific purposes. In this regard, Ionescu et al. inhibited nerve activity and subsequently eliminated impulse propagation in muscle fibers by using 1 μM Tetrodotoxin (TTX) in a chamber containing nerve cells, which has been suggested as a confirmatory approach of NMJ formation. Conversely, in the current investigation, the use of GluR agonists is proposed as a stimulator of nerve cells activities. Moreover, the authors have recommended that genetic interventions such as viral vectors can be applied for genetic manipulation of nerve and muscle cells (116).

In the same year, another study was conducted on the NMJ modeling in a 3D structure by applying mouse ESCs-derived motor neurons and C2C12-derived skeletal muscles. In this study, a transgenic ESC line (*ChR*2^*H*134*R*2^-HBG3) was formed, which had a major contribution to muscle contraction within the NMJ structure. In order to confirm the formation of NMJ, α-bungarotoxin (αBTX) has been applied with the intention to inhibit light-induced muscle contractions. Additionally, the combination of optogenetic technology with ESC–derived MNs and C2C12-derived muscle cells had a great contribution to optical controlling of NMJ activity. The integration of mentioned technology with cells made it possible to determine whether the cells possess the selectivity of optogenic stimulation. To achieve this goal, the contractile activity of muscle cells was examined, which was performed by changing the diameter of the excitation light beam and stimulating specific locations of the cells (117).

In addition to the previously mentioned studies, researchers have recently evaluated the effects of rapamycin (as an mTOR inhibitor) on improving neuronal survival by developing a microfluidic model for ALS. In this context, in order to provide ESCs, as one of the main components of the microfluidic system in modeling ALS, a transgenic mouse model has been created that provided manifestations of ALS disease by expressing the ubiquitinated cytoplasmic TDP-43 as a motor neuron-degrading agent. In this study, after the differentiation of stem cells, the effect of rapamycin in both 2D and 3D culture media was investigated. Studies in 2D culture medium indicate that 1 μM rapamycin, through decreasing cytosolic ubiquitinated TDP-43 aggregates, enhanced neuronal survival rate in treated mutant motor neurons. However, the ubiquitinated TDP-43 aggregates could be observed in untreated mutant motor nerve cells. In order to obtain more accurate results, a 2D cell culture method was integrated with a 3D microfluidic platform, in which the rapamycin gradient was established to affect mutant cells. In this context, the results of the study indicate that due to the threshold dose of rapamycin, no significant effect was reported on the viability of mutant cells at a concentration <0.2 μM. In contrast, a decrease in neuronal survival rate was observed at concentrations above 1.4 μM of rapamycin treatment compared to the effect of low concentrations on mutant cells. However, a significant increase in the viability of mutant motor neurons was reported in a concentration range from 0.4 to 1 μM compared to untreated mutant cells. Therefore, according to the findings of this study, it can be concluded that rapamycin can have a significant impact on the survival of motor neurons in ALS (118).

However, research in this area was not limited to the use of rodent stem cells. In recent years, hiPSC has attracted attention as a valuable candidate used in ALS-on-a-chip studies. In this context, Osaki et al. developed an ALS-on-a-chip, one able to recapitulate the human functional NMJ structure within microfluidic chips by creating the excitotoxicity of the motor nerve cells via excess glutamic acid treatment. In this study, to mimic the structure and function of human NMJ, sALS patient iPSC–derived motor neurons spheroids with a heterozygous G298S TDP43 mutation and iPSC-derived muscle in combination with optogenetic technology were employed. In this study, to ensure the accuracy of the applied method, the NMJ structure made from hESC-derived NSC spheroids along was considered a control model along with iPSC–derived cells model. In comparison between two NSC spheroids, significant points are concluded. For instance, iPSC–derived MN spheroids expressed less *islet1, ChAT, SMI-32*, and *Synapsin I* compared to hESC-derived MN spheroids. While the availability of NF-κB signaling pathway molecules in iPSC–derived MN is more than hESC-derived MN, which ultimately causes adverse effects on skeletal muscle cells. Additionally, the observations imply that iPSC–derived MN spheroids specifically experience a reduction in the size of the soma, augmentation in synaptic dysfunction, and programmed cell death. However, both iPSC and ESC-derived spheroids share several common formation and growth features (112).

In addition to the above, in 2019, Bauer et al. published a paper in which they developed an NMJ-on-a-chip model by applying pseudo-organoids of the human iPSCs-derived MNs and human myotubes. It is noteworthy to mention that this is the first set of analyses that investigated the impact of the glycoprotein-deleted rabies virus in the evaluation of NMJ functionality. In this experiment, the monosynaptic rabies virus tracing, also known as monosynaptic retrograde tracing, has been applied in which the viral DNA along with helper constructs (e.g., pCAGYTB24 or pAAV-CMV-TVAmCherry-2A-oG) is transferred into a myotube using electroporation technique. After this process, myotubes are able to express TVA receptors and rabies virus glycoproteins. In the next step, depending on the type of helper constructs, myotubes become infected with EnvA-ΔG-RV-mCherry or EnvA-ΔG-RV-GFP. As a result, the myotubes, as virus-infected initiated cells, retrogradely transmit the virons through the synapses to the MNs and infect them as well. Because the transferred genes contain fluorescent markers, the map of viral infection of neurons can be assessed by fluorescent assays, which are the verification of NMJ functionality. In addition to monosynaptic retrograde tracing, alpha-bungarotoxin, as the inhibitor of postsynaptic nicotinic acetylcholine receptors has been applied which has a crucial role in determining the contractile activity of myotubes. Furthermore, the use of immunostaining assay has also been examined as an effective approach to confirm the connection between myotubes and MNs in the suggested model (119).

In addition to disease modeling, researchers hope that OOC technology promise a bright future in the field of preclinical testing and drug toxicity experiments for various diseases, especially ALS. The micro-physiological chips, as a novel *in vitro* biomimetic systems, can hold considerable potential to address some of the factors involved in the failure of drug discovery and development pipelines in diseases such as ALS. For instance, iPSC technology is one of the practical features of micro-physiological chips that can greatly contribute to drug screening. Additionally, the transition of drug compounds through the blood-brain and blood-spinal cord barriers can be examined in these chips, provided that iPSC-derived endothelial cells be cultured in the compartment in which MN cells have been embedded. Therefore, according to the advantages of OOC technology, there is a great opportunity for examining a wide spectrum of drug candidates through micro-physiological biomimetic chips, which can be a big step toward discovering new approaches with higher therapeutic potential for patients.

Regarding NMJ dysfunctionality, different drugs such as rapamycin, bosutinib, reparixin, sunitinib, glutamic acid, acetylcholine, αBTX, and TTX have been examined via OOC technology. It should be mentioned that there is a need for the mentioned drugs to follow certain principles in order to be examined in connection with the NMJ structures designed within microfluidic chips. For example, before using drugs, concentrations should be determined according to parameters including IC50, the length of MN neurite, and the amount of myosin heavy chain expression in the muscle cells. In addition, the duration of drug administration should be considered according to the drug pharmacokinetic parameters and cell reactions to drugs (90).

## Conclusion and Future Perspective

The nervous system is considered the most organized and complex system of the body (120), which originates from a subtype of progenitor cells named neural stem cells (NSCs) (121) and coordinates the vital functions of the human body through a complex network of neurons (120). Over the past decade, the world has seen a growing number of patients with nerve injuries or disorders related to the nervous system (122). Despite considerable efforts toward applying various therapeutic approaches such as cell-based therapies for nerve damages (122–128), there remain challenges in improving nerve regeneration and treating nerve disorders and injuries (129). Additionally, notwithstanding the many efforts in the field of neuroscience, scientists have still not been to succeed extensively in the drug discovery and development platforms. The shortage of sufficient understanding of the mechanisms underlying neurological disorders is among the main challenges which should be noted (130). Additionally, a variety of *in vitro* and *in vivo* models are applied to mimic ALS mechanisms. However, the current models are not able to demonstrate appropriately the complexities of the human nervous system, which has posed difficulties to translational medicine (131). To meet the challenges, many efforts have been made to find and create innovative high-performance ALS modeling methods, that one of which is the OOC technology (114).

The emergence of OOC promises the dawn of a new era in the progression of disease modeling, which can provide the opportunities to pave the way for achieving deep knowledge of various diseases and discovering therapeutic approaches (132). But it is noteworthy mentioning that OOC as cutting-edge technology is in its infancy, and along with many significant advantages compared to other modeling methods, still faces challenges. For instance, no special attention is paid to the role of immune system components along with the main cells involved in microfluidic modeling systems, which can pose problems in accurate drug screening. Hence, this critical ignored aspect of biological function should be taken into consideration to avoid errors in drug discovery and development (133). Additionally, Polydimethylsiloxane, called PDMS is the most common material used in the fabrication of microfluidic chips. Although this polymeric material is applied as a substrate for establishing *in vivo* environment, the thickness of this layer in micro-physiological systems is much more than physiological conditions (88). Moreover, the PDMS structure is permeable to small hydrophobic molecules such as fluorescent dyes. Hence, these molecules are easily absorbed by the PDMS, which ultimately interferes with light tracking. Of interest is that recently, covering the PDMS with polytetrafluoroethylene (PTFE) has been proposed as a practical solution, which can have a significant contribution to increasing the sensitivity of optical detecting (134). Another drawback of OOC systems is the formation of bubbles in microfluidic channels, which is considered as a barrier to creating a suitable chip in modeling diseases (135). Although studies show that these bubbles can lead to cell damages by causing mechanical stress on cells, it could be an effective procedure in mimicking organs dysfunctionality such as cellular-level lung injuries and respiratory diseases (136). Microbial contamination of chips is another challenge in working with this technology that should be given a lot of attention. Otherwise, the output of the test is compromised.

In addition, the presence of an extracellular matrix is one of the requirements for cell attachment to the system. In this regard, simple ECM or a thin layer of the matrix may be used in OOC modeling. However, the ECM materials gradually lose their quality and degrade as time goes on. Therefore, cell survival runs into difficulties. Moreover, creating robust long-term microfluidic cell culture in OOC systems is another obstacle to fabricating a suitable chip (135). However, according to studies, periodic delivery of the medium with fast pulses has been reported as an effective approach in homogeneous cell culture which can lead to an increase in the viability of the cells (137).

In addition to the above, the regulation of cell density has been one of the problems in the development of microfluidic systems in the past. However, a study using interdigital electrode structures (IDES) recently examined the resulting impedance and recorded cell proliferation rates using feedback controllers to maintain and stabilize cell density, which can be considered a great success in this field (138). Furthermore, industry reception is another important issue in the field of chip technology. Despite the use of OOC technology leading to a significant reduction in the cost of drug production (139), micro-physiological systems entail high production costs. It is therefore imperative to use cheaper components or to use the previous components several times in the future. Additionally, in the physiological conditions of the body, the interaction between different organs and systems play a significant role in complicating human biological conditions. Accordingly, researchers are seeking for making connections between different types of organ on chip models to achieve multi-organ or human on a chip micro-physiological systems. While this idea could revolutionize the future of disease modeling and drug discovery, it is clear, the more complex the system, the greater the problems. Therefore, constructing multi-organ systems on a chip still needs more studies to be able to reach its turning point in the future. These are just some of the problems with the OOC biomimetic technology, which proves that there is still a long way to go before holding great potential of OOC as a common technology for applying in biomedical studies in the future (88).

## Author Contributions

BA, SK, ZR, AT-B, FR, HA, and BL contributed to the preparation of the draft of the manuscript. All authors read and approved the final manuscript.

## Conflict of Interest

The authors declare that the research was conducted in the absence of any commercial or financial relationships that could be construed as a potential conflict of interest.

## Publisher's Note

All claims expressed in this article are solely those of the authors and do not necessarily represent those of their affiliated organizations, or those of the publisher, the editors and the reviewers. Any product that may be evaluated in this article, or claim that may be made by its manufacturer, is not guaranteed or endorsed by the publisher.
